# ZnO nanoparticles sensitized by CuInZn*_x_*S_2+_*_x_* quantum dots as highly efficient solar light driven photocatalysts

**DOI:** 10.3762/bjnano.8.110

**Published:** 2017-05-17

**Authors:** Florian Donat, Serge Corbel, Halima Alem, Steve Pontvianne, Lavinia Balan, Ghouti Medjahdi, Raphaël Schneider

**Affiliations:** 1 CNRS and Université de Lorraine, Laboratoire Réactions et Génie des Procédés (LRGP), CNRS UMR 7274, 1 rue Grandville 54001 Nancy, France; 2 CNRS and Université de Lorraine, Institut Jean Lamour (IJL), UMR CNRS 7198, BP 70239, 54506 Vandoeuvre-lès-Nancy Cedex, France; 3 Institut de Science des Matériaux de Mulhouse (IS2M), CNRS UMR 7361, 15 rue Jean Starcky, 68093 Mulhouse, France

**Keywords:** heterojunction, photocatalysis, quantum dots, reactive oxygen species, singlet oxygen, ZCIS quantum dots, ZnO

## Abstract

Alloyed CuInZn*_x_*S_2+_*_x_* (ZCIS) quantum dots (QDs) were successfully associated to ZnO nanoparticles by a thermal treatment at 400 °C for 15 min. The ZnO/ZCIS composite was characterized by TEM, SEM, XRD, XPS and UV–vis absorption spectroscopy. ZCIS QDs, with an average diameter of ≈4.5 nm, were found to be homogeneously distributed at the surface of ZnO nanoparticles. ZCIS-sensitized ZnO nanoparticles exhibit a high photocatalytic activity under simulated solar light irradiation for the degradation of Orange II dye (>95% degradation after 180 min of irradiation at an intensity of 5 mW/cm^2^). The heterojunction built between the ZnO nanoparticle and ZCIS QDs not only extends the light adsorption range by the photocatalyst but also acts to decrease electron/hole recombination. Interestingly, the ZnO/ZCIS composite was found to produce increased amounts of H_2_O_2_ and singlet oxygen ^1^O_2_ compared to ZnO, suggesting that these reactive oxygen species play a key role in the photodegradation mechanism. The activity of the ZnO/ZCIS composite is retained at over 90% of its original value after ten successive photocatalytic runs, indicating its high stability and its potential for practical photocatalytic applications.

## Introduction

Over the last twenty years, the field of photocatalysis has attracted wide attention due to the environmental and energy crisis. Photocatalysis is considered to be a green technology, allowing the degradation of numerous contaminants both in water and air [1–5] and can also be used to produce energy vectors such as hydrogen from water [6–8]. Due to their strong catalytic activity, reasonable photo- and chemical stability, and weak toxicity, TiO_2_ and ZnO semiconductors are the most commonly used photocatalysts. However, their large bandgap (≈3.2–3.3 eV) restricts light activation to the UV range (which accounts for only ≈4% of the solar spectrum) for the generation of the charge carriers responsible for the surface redox reactions.

To improve the efficient use of solar light, visible-light-responsive photocatalysts should be developed. This can be achieved by combining TiO_2_ or ZnO with narrow bandgap semiconductors such as quantum dots (QDs), where generally cadmium or lead chalcogenides are applied [9]. Due to the close interfacial contact between the semiconductors, the electronic structures of ZnO or TiO_2_ are strongly coupled to those of the QDs [10–12]. Heterostructured photocatalysts such as ZnO/CdS or TiO_2_/CdS or TiO_2_/PbS exhibit extended light absorption and improved photoreactivity due to the promoted separation of photo-induced charge carriers [13–19]. However, QDs such as CdS or PbS contain highly toxic elements, which severely restricts their use.

In recent years, I–III–VI_2_ group semiconductors such as CuInS_2_ (CIS) have emerged as environmentally friendly alternatives to Cd- or Pb-based QDs [20]. CIS QDs can be alloyed with ZnS to generate CuInZn*_x_*S_2+_*_x_* (ZCIS) nanocrystals with high absorption coefficients and bandgaps covering almost the whole visible spectrum [21–23], which is of high interest for solar-driven photocatalysis. CIS or ZCIS QDs have recently been associated with TiO_2_ [24–31] and ZnO [32–41] nanoparticles to develop materials for various photonic applications such as photoelectrodes, solar cells or photocatalysts. Despite the photosensitizing properties of ZCIS QDs and their increased stability compared to CIS cores, ZCIS QDs have yet to be combined with ZnO to develop photocatalysts. Moreover, ZnO/CIS composites prepared for the photocatalytic degradation of pollutants require a relatively large quantity of CIS nanocrystals to be complete (20 wt % CIS QDs relative to ZnO) [34,37]. When the ZCIS/ZnO weight ratio was decreased to ≈7%, the photodegradation never exceeded 80% even using a light source generating UV-A and UV-B radiation.

In this paper, we report first the successful preparation of a ZnO/ZCIS heterostructured photocatalyst using commercial ZnO nanoparticles and only 2.5 wt % of ZCIS QDs. The high photocatalytic activity of this material for the degradation of Orange II dye under simulated solar light irradiation was demonstrated. The ZnO/ZCIS photocatalyst was found to possess high stability and could be reused at least ten times without significant loss of activity. Additionally, it was found to be only weakly sensitive to interfering substances such as salts present in the aqueous solution. A mechanism for the degradation pathways mediated by the ZnO/ZCIS catalyst is proposed. Interestingly, hydrogen peroxide, H_2_O_2_, and singlet molecular oxygen, ^1^O_2_, were found to play a key role in the oxidation of Orange II.

## Experimental

### Materials

Indium acetate (In(OAc)_3_, 99.99%, Sigma), zinc acetate (Zn(OAc)_2_, 99.99%, Sigma), copper iodide (CuI, 99.999%, Sigma), dodecanethiol (DDT, >98%, Sigma), oleylamine (OA, 70%, Sigma), 1-octadecene (ODE, 90%, Sigma), zinc oxide (ZnO, 99%, Alfa Aesar), disodium terephthalate (DST, 99+%, Alfa), nitrotetrazolium blue chloride (NBT, >98%, Sigma), leuco crystal violet (LCV, 99%, Sigma), enzyme horseradish peroxidase (HRP, type II, Sigma), singlet oxygen sensor green (SOSG, Molecular Probes), Rose Bengal (RB, 95%, Sigma) and chloroform (>99%, Carlo Erba) were used without further purification.

### ZCIS QD synthesis

ZCIS QDs were synthesized using the Zn(OAc)_2_-OA complex for the introduction of a ZnS shell covering the CIS QD core according to the synthetic procedure we recently developed [23], with slight modifications. Briefly, CuI (0.14 mmol), In(OAc)_3_ (0.2 mmol) and ODE (16 mL) were loaded into a 100 mL three-neck flask under argon flow. The reaction mixture was further degassed by heating at 75 °C under vacuum for 20 min and then backfilled with argon. DDT (8.3 mmol) was subsequently injected and the temperature of the reaction solution was slowly raised to 210 °C. The reaction time was set to 20 min. In a separate flask, Zn(OAc)_2_ (1.8 mmol) was dissolved in an OA/ODE mixture (4.1 mmol/10 mmol, respectively) at 100 °C under nitrogen. The solution was maintained at 80 °C after dissolution of the reagents.

After 20 min of CIS core growth, a first portion of Zn^2+^-OA/ODE mixture (0.5 mL) was injected into the crude core solution maintained at 210 °C under argon flow. This step was repeated every 15 min (total injected volume: 4.5 mL). Finally, the reaction was cooled down to room temperature and 5 mL of toluene were added. ZCIS QDs were precipitated with ethanol and the mixture was centrifuged. The supernatant was removed and the solid was redispersed in toluene, and reprecipitated by adding ethanol. The centrifugation and precipitation procedure was repeated eight times for the purification of the ZCIS QDs.

### Preparation of the ZnO/ZCIS photocatalyst

For the synthesis of the ZnO/ZCIS catalyst used in this work, 2.5 mg of dried ZCIS QDs were dispersed in 10 mL CHCl_3_. 100 mg of commercial ZnO nanoparticles, preliminary calcined at 450 °C for 3 h, were then added and the mixture which was magnetically stirred at room temperature until complete evaporation of CHCl_3_. The powder obtained was then heated at 400 °C for 15 min to build the heterojunction between ZnO nanoparticles and ZCIS QDs.

### Photocatalytic performance test

A 100 mL Pyrex glass flask was used as a batch reactor. All experiments were performed at ambient temperature (20 ± 2 °C). In a typical photocatalytic experiment, 30 mg of the ZnO/ZCIS catalyst were added to 50 mL of an aqueous Orange II solution (10 mg/L) in the flask which was open to the air. The mixture was magnetically stirred for 30 min in the dark to reach a thorough adsorption/desorption equilibrium and then exposed to simulated solar light irradiation using Sylvania Luxline plus T5/FHO 24 W neon tubes under continuous stirring. Using a radiometer, the light intensity was estimated to be 5 mW/cm^2^. At regular intervals, 2 mL sample aliquots were withdrawn from the reaction flask and centrifuged (15,000 rpm for 2 min) to remove the photocatalyst. The photodegradation progress was monitored by measuring the UV–vis absorption of Orange II at λ_max_ = 485 nm.

### Detection of ^•^OH radicals: DST assay

The production of **^•^**OH radicals by ZnO and the ZnO/ZCIS photocatalyst was measured by using disodium terephthalate (DST). DST turns into fluorescent 2-hydroxyterephthalate, 2-OH-DST (λ_em_ = 428 nm) upon reaction with an **^•^**OH radical. The ZnO or ZnO/ZCIS photocatalyst (5 mg) was dispersed in 100 mL of water. 1 mL of this dispersion was mixed with 1 mL of a 0.1 M aqueous DST solution before irradiation with a Hg–Xe lamp (light intensity, 200 mW/cm^2^) for various durations (0, 1, 5, 10, 15 and 30 min). Then, 1 mL of 1 M NaOH solution was added and the mixture was incubated for 50 min at room temperature in the dark. The photoluminescence spectra were recorded in order to estimate the formation of 2-OH-DST (λ_ex_ = 300 nm). The quantification of ^•^OH production was conducted according to the method we recently described [42].

### Detection of O_2_^•−^ radicals: NBT assay

The production of superoxide O_2_^•−^ radicals was measured using the nitroblue tetrazolium (NBT). The reduction of the yellowish NBT into purple formazan derivatives results in an increase of the absorbance between 450 and 700 nm. The ZnO or ZnO/ZCIS photocatalyst (5 mg) was dispersed in a 1:1 water/DMSO mixture (100 mL). This mixture was saturated with oxygen. NBT (8 mg) was added to the photocatalyst dispersion under light protection. Then the solution was irradiated with a Hg–Xe (light intensity, 50 mW/cm^2^) for various durations (0, 5, 15, 30, 45 and 60 min). The formazan derivatives formed were quantified by recording the absorbance spectra of formazan according to our previous report [42].

### Detection of H_2_O_2_: Crystal violet assay

The production of H_2_O_2_ by ZnO and ZnO/ZCIS photocatalysts was measured by using leuco crystal violet (LCV), which forms a violet cation (LCV^+^) absorbing at 596 nm in the presence of horseradish peroxidase (HRP) and H_2_O_2_. For the preparation of the HRP solution, 10 mg of HRP were dissolved in 10 mL of water. For the LCV solution, 5 mg of LCV were dissolved in 10 mL of 0.5% HCl solution. The acetate buffer was prepared by mixing equal volumes of sodium acetate (2 M) and acetic acid followed by pH adjustment at 4.5 using glacial acetic acid.

A dispersion of photocatalyst (5 mg) in 100 mL of water was irradiated during 45 min with a Hg–Xe source (light intensity, 50 mW/cm^2^). 200 µL of the irradiated solution were added to 9.8 mL of distilled water. Next, 1 mL of the LCV solution and 0.5 mL of the HRP solution and 5 mL of acetate buffer were added. The mixture was stirred in the dark at room temperature for 60 min. The concentration of H_2_O_2_ was determined by measuring the absorption of LCV^+^ at 596 nm followed by comparison to a calibration curve made with standard solutions of H_2_O_2_ [42].

### Detection of ^1^O_2_: SOSG assay

The production of ^1^O_2_ was measured by using the Singlet Oxygen Sensor Green (SOSG) probe. 100 µg of SOSG were dispersed in 6.6 mL of methanol in order to prepare a stock solution of SOSG (25 µM). ZnO or ZnO/ZCIS photocatalysts (5 mg) were dispersed in 100 mL of water and the solution was saturated with oxygen during 1 h. 2.5 mL of the dispersion and 100 µL of SOSG solution were introduced in a cuvette for irradiation under Hg–Xe (light intensity, 50 mW/cm^2^) for various durations (0, 1, 3, 6, 9, 12 and 15 min). The production of ^1^O_2_ was evaluated by measuring the photoluminescence of SOSG–endoperoxide (SOSG-EP) (λ_ex_ = 480 nm and λ_em_ = 525 nm) [42].

### Instruments and characterization

Transmission electron microscopy (TEM) investigations were performed with a JEOL ARM 200F – Cold FEG TEM/STEM (point resolution 0.19 nm in TEM mode and 0.078 nm in STEM mode) fitted with a GIF Quatum ER. High-resolution TEM (HR-TEM) imaging was performed with a JEOL ARM 200F – Cold FEG (point resolution 0.19 nm) fitted with a GIF Quatum ER. For each sample, one drop of a dispersed solution was deposited on holey carbon grids and imaged. Scanning electron microscopy (SEM) pictures were prepared using a JEOL scanning electron microscope JSM-6490 LV. The X-ray diffraction (XRD) data were collected from an X'Pert MPD diffractometer (Panalytical AXS) with a goniometer radius 240 mm, fixed divergence slit module (1/2° divergence slit, 0.04 rd Sollers slits) and an X'Celerator as a detector. The powder samples were placed on a silicon zero-background sample holder and the XRD patterns were recorded at room temperature using Cu Kα radiation (λ = 0.15418 nm). X-ray photoelectron spectroscopy (XPS) analyses were performed on a Gammadata Scienta (Uppsala, Sweden) SES 200-2 spectrometer under ultra-high vacuum (*P* < 10^−9^ mbar). The spectrometer resolution at the Fermi level is about 0.4 eV. The depth analyzed extends up to about 8 nm. The monochromatized Al Kα source (1486.6 eV) was operated at a power of 420 W (30 mA and 14 kV) and the spectra were acquired at a take-off angle of 90° (angle between the sample surface and photoemission direction). During acquisition, the pass energy was set to 500 eV for wide scans and to 100 eV for high-resolution spectra. CASA XPS software (Casa Software Ltd, Teignmouth, UK, http://www.casaxps.com) was used for all peak fitting procedures and the areas of each component were modified according to classical Scofield sensitivity factors. The initial and final total organic carbon (TOC) content was determined using a Shimadzu TOC-V_CSH_ analyzer to evaluate the degree of photomineralization. The zeta potential (ζ) of the ZnO/ZCIS nanoparticles was determined using a Malvern Zetasizer Nano ZS instrument.

All the optical measurements were conducted at room temperature (20 ± 1 °C) under ambient conditions. The absorption spectra of liquid samples were recorded on a Thermo Scientific Evolution 220 UV–visible spectrophotometer. The photoluminescence (PL) spectra were recorded on a Horiba Fluoromax-4 Jobin Yvon spectrofluorimeter. The diffuse reflectance spectra (DRS) were recorded on a Shimadzu 2600 UV–vis spectrophotometer. BaSO_4_ powder was used as a standard for baseline measurements and spectra were recorded in a range of 250–1400 nm.

## Results and Discussion

### ZnO/ZCIS photocatalyst synthesis and characterization

**Figure 1:**
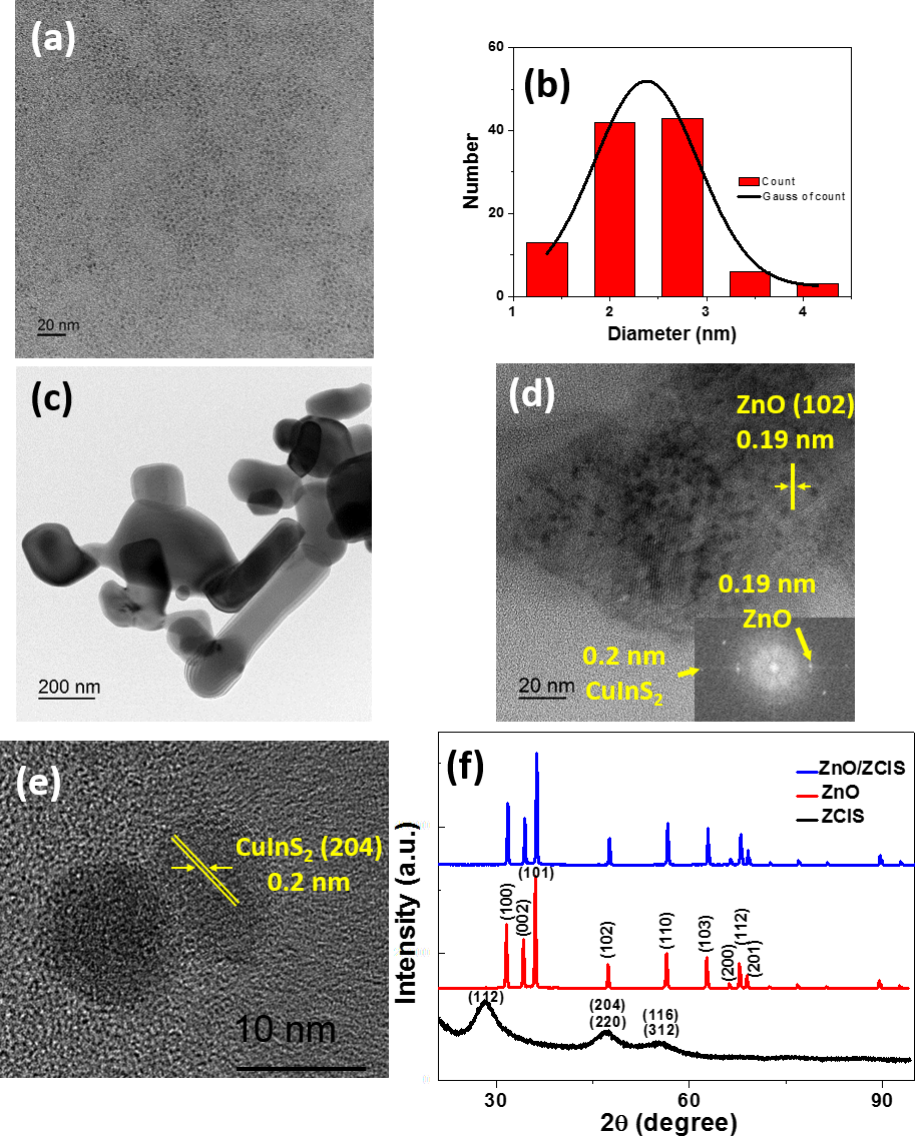
(a) TEM image of ZCIS QDs and (b) the corresponding size distribution. TEM and HR-TEM images of (c) ZnO nanoparticles and (d,e) the ZnO/ZCIS composite. (f) XRD patterns of ZCIS QDs, ZnO nanoparticles and of the ZnO/ZCIS composite.

QDs with a CIS core were prepared by a high temperature decomposition method in the noncoordinating solvent 1-octadecene using In(OAc)_3_, CuI and dodecanethiol (DTT) as starting materials. DDT serves both as the sulfur source and as the stabilizing ligand for the nanocrystals. A Cu/In ratio of 0.7 was used for the synthesis because Cu-deficient CIS QDs exhibit higher PL quantum yields than those prepared with a stoichiometric Cu/In ratio [43]. To improve the PL efficiency, a ZnS shell was coated on CIS core QDs using the Zn(OAc)_2_-OA complex [23]. The obtained ZCIS QDs absorb light until ca. 700 nm and their PL maximum is located at ca. 620 nm (Supporting Information File 1, Figure S1). The PL quantum yield of these red-emitting dots is 50% in toluene and their bandgap, estimated through the Tauc’s plot, is 2.1 eV (Supporting Information File 1, Figure S2). Using XPS, the Cu/In/Zn/S atomic ratio of the purified ZCIS QDs was determined to be 1.0:1.43:8.54:20.0, indicating that the Cu/In ratio is very close to that used for the synthesis (0.7) and that S is in marked excess compared to the stoichiometry of the quantum dots due to the presence of DDT at their periphery (Supporting Information File 1, Figure S3). A TEM image of ZCIS QDs shows that they are well-dispersed and that their average diameter is 2.3 ± 0.5 nm (Figure 1a,b). Commercial ZnO nanoparticles used in this study are heterogeneous in size and morphology and the sample contains spherical and ellipsoidal nanoparticles as well as nanorods (Figure 1c). To build the heterojunction between ZCIS QDs and ZnO nanoparticles, the nanoparticles were first dispersed in chloroform and stirred at room temperature without any protection until complete evaporation of the solvent. Next, the ZnO/ZCIS powder was heated at high temperature to decompose the DDT ligand covering ZCIS QDs and create a heterojunction between ZnO and ZCIS QDs that facilitates the electron transport process.

In preliminary experiments, we varied the amount of ZCIS QDs associated to ZnO (0.5, 1, 2.5, 5, 10 and 20 wt %), the calcination time (15 min, 1, 2 or 12 h) and the temperature (100, 200, 300 and 400 °C). ZnO nanoparticles do not absorb in the visible range (λ > 400 nm). Once ZCIS QDs are associated to ZnO nanoparticles, a noticeable increase of absorption in the visible region, centered at ≈675 nm and dependent on the loading in ZCIS QDs is observed (Supporting Information File 1, Figure S4). An increased photo-response of ZnO/ZCIS composites in the visible region compared to ZnO can thus be expected.

With the aim of investigating the photocatalytic performance both under solar and visible light illumination (light intensity = 5 mW/cm^2^) of all the ZnO/ZCIS composites prepared, we selected Orange II dye as a model contaminant because this dye is not a photosensitizer (in contrast to Methylene Blue or Rhodamine which promote photocatalytic degradation). Prior to any light irradiation, the reaction mixture was stirred in the dark for 30 min to reach adsorption/desorption equilibrium. Blank experiments in the absence of irradiation but in the presence of the ZnO/ZCIS catalysts demonstrated that no significant change in the Orange II concentration was observed. Under both irradiation conditions, optimal photodegradation results were obtained by using 2.5 wt % ZCIS QDs relative to ZnO, and when the heterojunction between these materials was created by a thermal treatment at 400 °C for 15 min. The optimized ZnO/ZCIS (2.5 wt %) photocatalyst will be used in all further studies. Before the complete evaluation of the photocatalytic activity, the ZnO/ZCIS material was characterized by XRD, TEM, SEM and XPS.

TEM images show that ZCIS nanocrystals were dispersed at the surface of ZnO nanoparticles and that their average diameter slightly increases (≈4.5 nm) after the thermal treatment at 400 °C (Figure 1d). No aggregation of ZCIS particles was observed from the TEM image after this annealing process. The HR-TEM image and the interplanar spacings measured confirm the co-existence of ZnO in the hexagonal phase and ZCIS particles with a tetragonal structure, suggesting that the heterojunction between the two semiconductors has been constructed (Figure 1e). XRD patterns of ZCIS QDs, ZnO and ZnO/ZCIS particles are shown in Figure 1f. For ZCIS QDs, the main peaks appear at 2Θ values of 28.21, 47.09 and 55.28° and can be indexed to the (112), (204)/(220) and (116)/(312) planes of the tetragonal chalcopyrite-like structure of CIS (Roquesite, JCPDS No 47-1372). For ZnO, the peaks at 2Θ values of 31.79, 34.46, 36.29, 47.56, 56.62, 62.87, 66.41, 67.95 and 69.09° correspond to the (100), (002), (101), (102), (110), (103), (200), (112) and (201) crystal planes of wurtzite-type ZnO (JCPDS No 36-1451). Reflections of ZnO remain unchanged after association with ZCIS QDs, indicating that its crystalline structure was not affected. The XRD peaks of the QDs could not be detected in the ZnO/ZCIS composite due to the low content of QDs in the composite (2.5 wt %).

**Figure 2:**
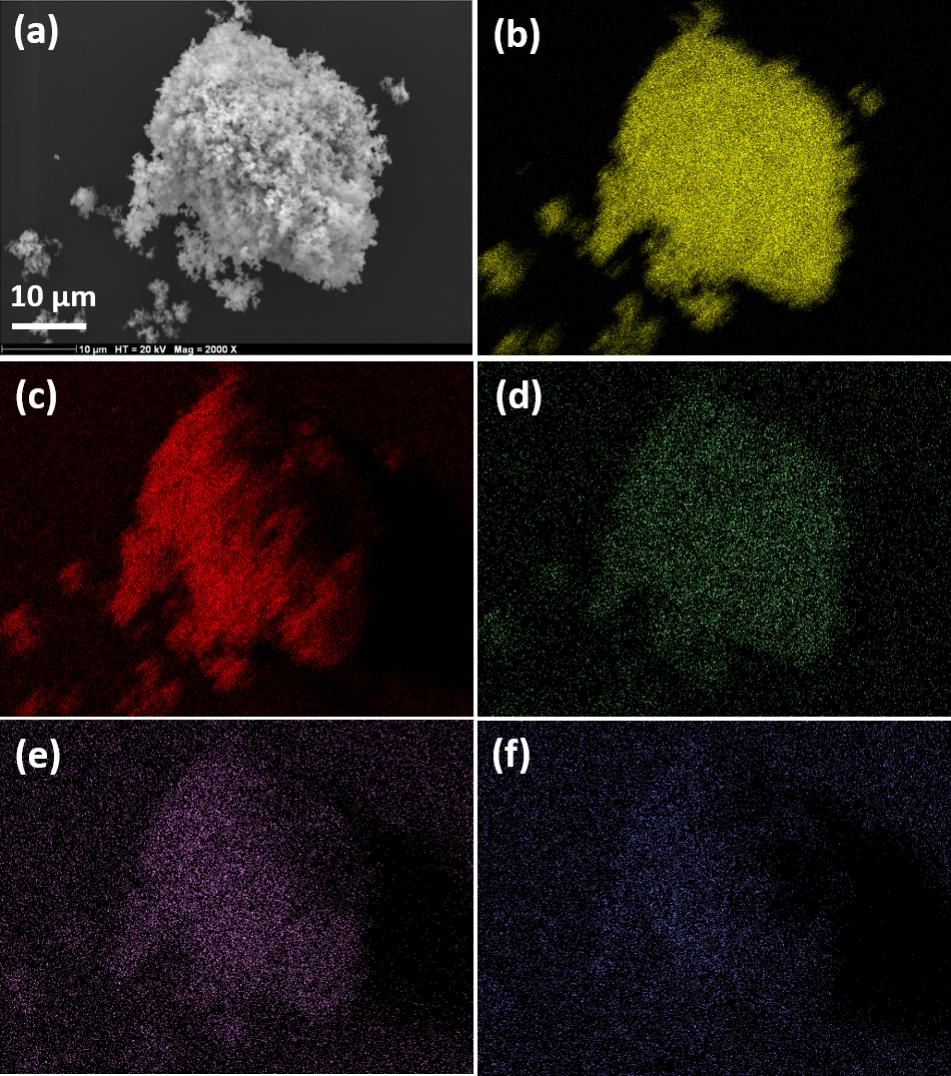
(a) SEM image of the ZnO/ZCIS composite. Elemental mapping of the ZnO/ZCIS composite heated at 400 °C for 15 min showing the presence of (b) Zn, (c) O, (d) Cu, (e) In and (f) S elements.

A typical SEM image of the ZnO/ZCIS composite is shown in Figure 2a. ZnO nanoparticles with a diameter of ≈0.2 µm can be observed, which is in accordance with TEM experiments. The association of ZCIS QDs with ZnO and the relative uniformity of the QD distribution at the surface of ZnO nanoparticles was demonstrated using energy dispersive X-ray spectroscopy (EDS) (Supporting Information File 1, Figure S5). Cu, In and S were present besides Zn and O and the elemental mapping indicates that these elements were homogeneously distributed at the surface of ZnO nanoparticles (Figure 2b–f).

**Figure 3:**
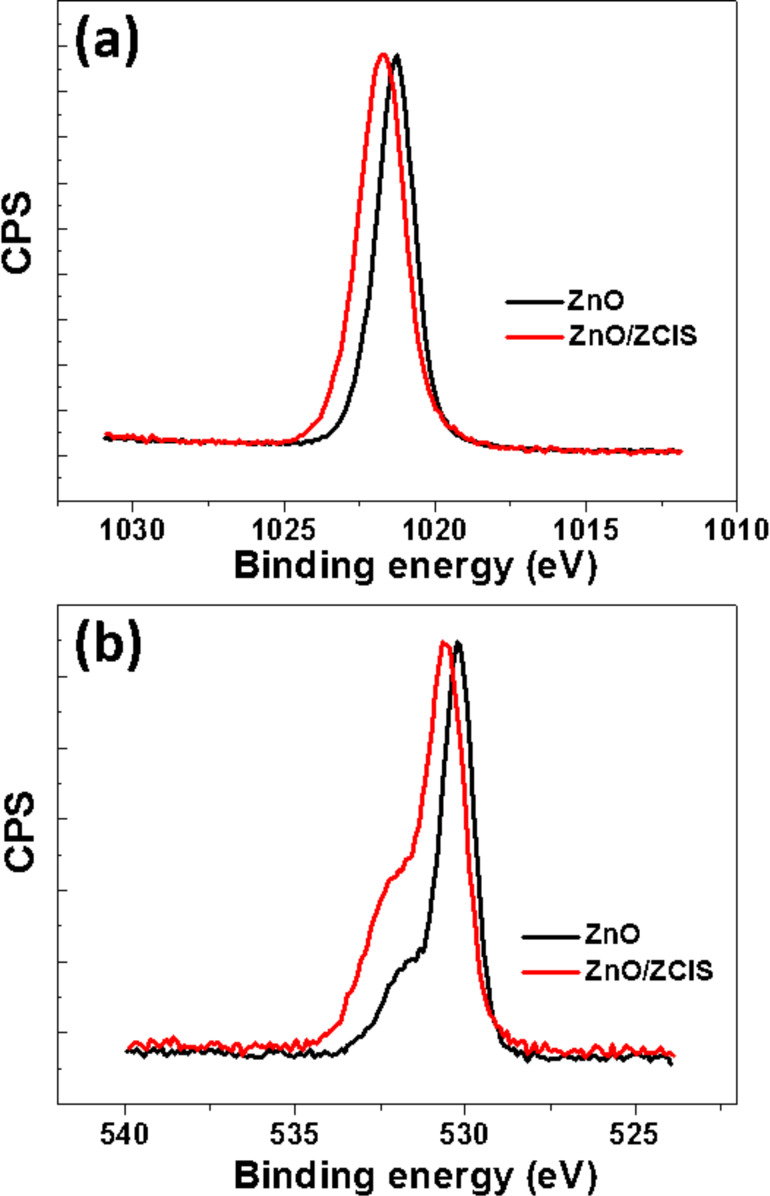
High-resolution XPS spectra of (a) Zn 2p_3/2_ and (b) O 1s in ZnO and in the ZnO/ZCIS composite.

XPS was further used to determine the binding energy of Zn 2p and O 1s in ZnO and ZnO/ZCIS particles and demonstrates the association of these materials (Figure 3). Compared to the binding energy of Zn 2p_3/2_ and O 1s in ZnO (1021.25 and 530.25 eV, respectively), a shift to higher energies was observed in the ZnO/ZCIS heterostructure (1021.77 and 530.67 eV for Zn 2p_3/2_ and O 1s, respectively), indicating the formation of new chemical bonds and the creation of an heterojunction between ZnO and ZCIS QDs.

Based on the previously described optical results (*E*_g_ (ZnO) = 3.25 eV and *E*_g_ (ZCIS) = 2.1 eV), the band edge positions of the valence band (VB) and conduction band (CB) of ZnO nanoparticles and ZCIS QDs were estimated using the Mulliken electronegativity theory [44–45]:

[1]
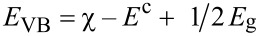


**Figure 4:**
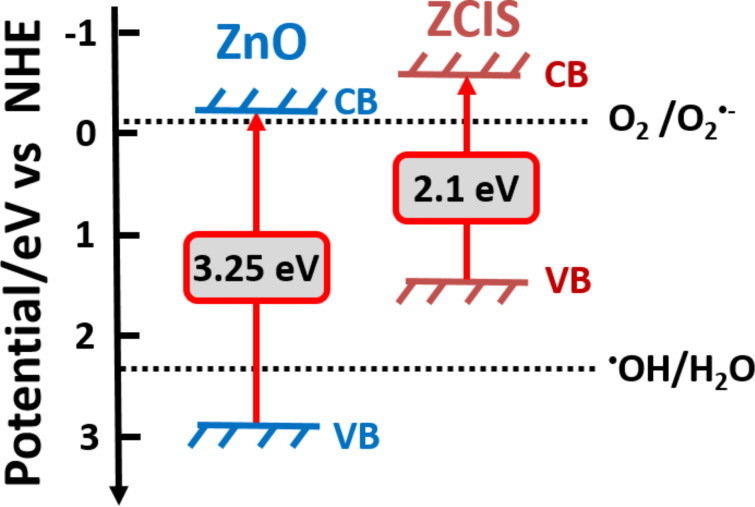
Band structure of ZnO and ZCIS QDs and redox potentials of O_2_/O_2_^•−^ and ^•^OH/H_2_O couples.

[2]
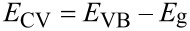


where χ is the absolute electronegativity of the semiconductor (expressed as the geometric mean of the absolute electronegativity of the constituent atoms) and *E*^c^ is the energy of free electrons on the hydrogen scale (4.5 eV). The χ values of ZnO and CuInZn*_x_*S_2+_*_x_* were determined to be 5.79 and 4.96 eV, respectively. Using Equation 1 and Equation 2, the *E*_VB_ values of ZnO and ZCIS QDs were estimated to be 2.91 and 1.51 eV, respectively. The relative positions of the CBs of ZCIS QDs and ZnO indicate that these materials exhibit a well-coupled band structure which should be favorable for the separation of photo-generated charge carriers and thus for photocatalytic experiments (Figure 4).

### Photocatalytic experiments

#### Influence of the Orange II concentration and catalyst loading

**Figure 5:**
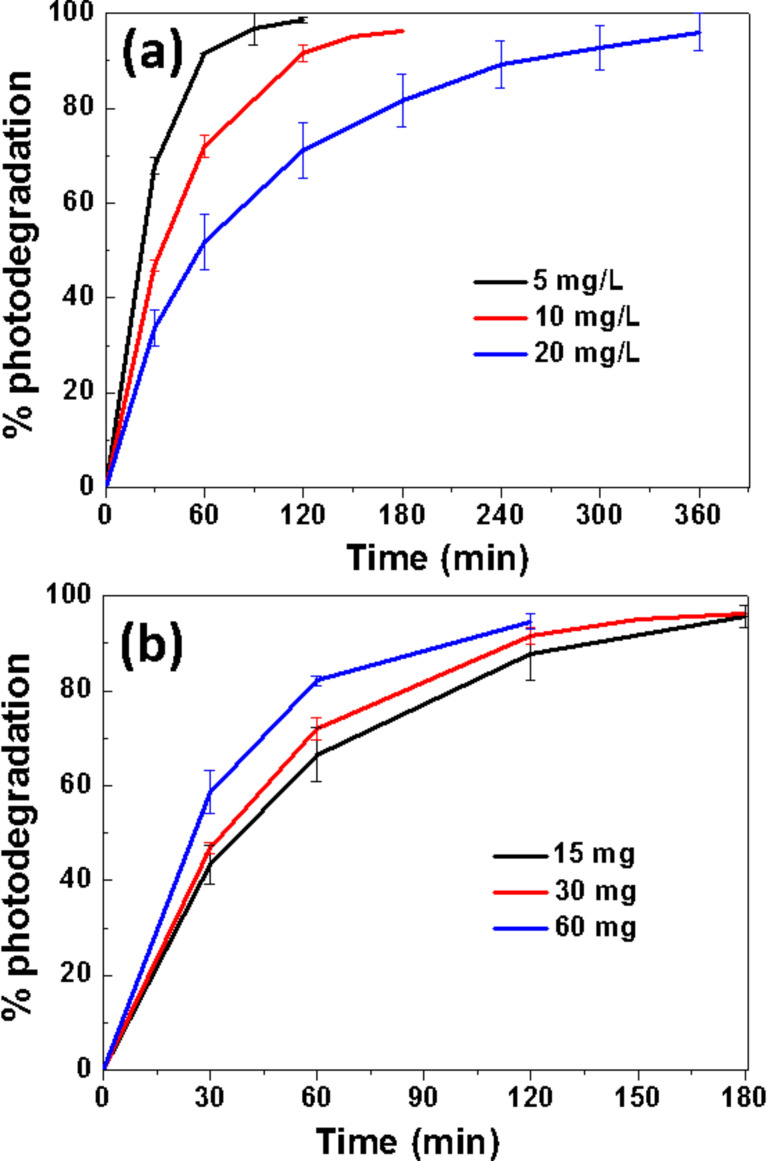
(a) Influence of the Orange II concentration and (b) of the ZnO/ZCIS catalyst loading on the photodegradation efficiency.

We first evaluated the effect of the initial Orange II concentration (5, 10 or 20 mg/L) on the photocatalytic degradation (Figure 5a). As can be seen, the percentage of photodegradation increases when the initial concentration of the dye decreases. Considering the initial (*C*_0_) and at time *t* (*C*) concentrations of Orange II, the ln(*C*_0_/*C*) plots show a linear relationship with the irradiation time, indicating that the photodegradation occurs via a pseudo-first-order kinetic reaction ln(*C*_0_/*C*) = −*kt*, where *k* is the photodegradation rate constant (min^−1^) (Supporting Information File 1, Figure S6). The *k* values were estimated to be 0.035, 0.018 and 0.008 min^−1^ for Orange II concentrations of 5, 10 and 20 mg/L, respectively. The decrease of the reaction rate with increasing concentration of dye is related both to the decrease of the probability of contact between the dye and the reactive oxygen species (ROS) generated at the surface of the ZnO/ZCIS catalyst and to the increased amount of incident photons absorbed by the dye (filter effect) and thus to the decreased amount of light available for the production of ROS.

The influence of the catalyst loading (15, 30 or 60 mg in 50 mL of solution) on the photocatalytic degradation of Orange II (used at 10 mg/L concentration) was also studied and the degradation profiles are shown in Figure 5b. After 30 or 60 min of solar light irradiation, the percentage of degradation increases linearly with the amount of catalyst due to the increased number of catalytically active sites (*k* values are 0.017, 0.018 and 0.022 min^−1^ for catalysts amounts of 15, 30 and 60 mg in 50 mL of the dye) (Supporting Information File 1, Figure S7). After 120 min of irradiation, the percentage of photodegradation leveled off due to the weak amount of Orange II remaining in solution. Based on these results, a photocatalyst concentration of 0.6 g/L was used throughout the present study.

Using dye and catalyst concentrations of 10 mg/L and 0.6 g/L, respectively, a complete decolorization of the aqueous solution was observed and the TOC decreased from 5.14 to 2.45 mg/L. This result shows that Orange II has not only been degraded but also efficiently mineralized using the ZnO/ZCIS photocatalyst. Noteworthy is also that when the photocatalytic reaction was extended up to 10 h, the TOC value decreased to 0.

#### Influence of pH and interfering species

**Figure 6:**
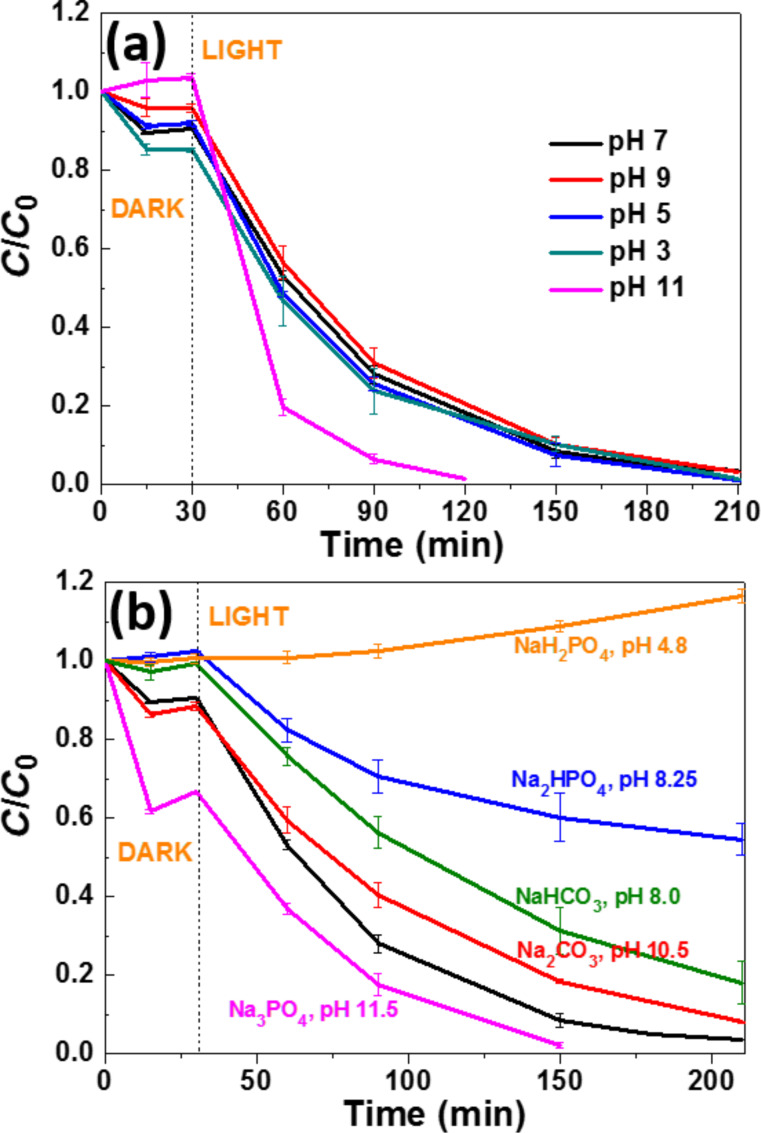
(a) Influence of pH and (b) of phosphates and carbonates on the photocatalytic activity of the ZnO/ZCIS composite (the black curve corresponds to the reference photocatalytic test conducted in water at pH 7).

The pH value is an important parameter when performing photocatalytic experiments because the amount of ROS generated upon illumination of the photocatalyst depends on pH [46]. We first investigated the influence of pH in the 3–11 range on the photocatalytic efficiency of the ZnO/ZCIS composite and by adjusting the pH with 0.01 M NaOH or HCl solutions. As shown in Figure 6a, in the 3 to 9 pH range, no marked influence of pH was observed and the complete bleaching of Orange II was achieved within 180 min (note that the adsorption of Orange II is slightly enhanced at pH 3, *C*/*C*_0_ = 0.83 compared to pH 5, 7 and 9). The *k* values determined were 0.022, 0.024, 0.018 and 0.018 min^−1^ at pH 3, 5, 7 and 9, respectively.

We next conducted photocatalytic experiments in 10 mM solutions of carbonate or phosphate salts that are commonly used to adjust the pH and enhance the dye fixation in textile fabrics (the pH values were varying from 4.8 for NaH_2_PO_4_ to 11.5 for Na_3_PO_4_) (Figure 6b). Na_2_CO_3_ and NaHCO_3_ reduced the photodegradation rates probably because these salts may adsorb at the surface of the ZnO/ZCIS catalyst and hinder the adsorption of the Orange II dye or consume ^•^OH radicals. The generated carbonate radicals exhibit a lower oxidation capacity than ^•^OH radicals (^•^OH + HCO_3_^−^ → CO_3_^•−^ + H_2_O and ^•^OH + CO_3_^2−^ → CO_3_^•−^ + ^−^OH) [47–48]. Unlike carbonates, Na_3_PO_4_ enhances the photodegradation efficiency probably due to the high pH value (11.5) of the solution. This result also indicates that Na_3_PO_4_ does not adsorb at the surface of the ZnO/ZCIS catalyst (electrostatic repulsion between PO_4_^3−^ ions and the photocatalyst at a pH value higher than the point of zero charge (pzc) of the ZnO/ZCIS catalyst, pzc = 8.35) and because the adsorption of Orange II in the dark is markedly enhanced. The photodegradation rate was markedly reduced in the presence of Na_2_HPO_4_ (pH 8.25) and even stopped when NaH_2_PO_4_ (pH 4.8) was used, indicating that these salts markedly adsorb on the ZnO/ZCIS catalyst and block the active surface sites and/or consume the ROS that are photo-produced.

The influence of other salts commonly found in wastewater such as Na_2_SO_4_, NaNO_3_, CaCl_2_, MgCl_2_ or NaCl was also investigated (all these salts were used at a 10 mM concentration). Neither the adsorption of Orange II at the surface of the ZnO/ZCIS catalyst nor its photodegradation were significantly affected by the presence of these salts (Supporting Information File 1, Figure S8). Noteworthy is also that chloride ions exhibit no hole scavenging activity (Cl^−^ + h^+^ → Cl^•^) and that the electron/hole separation generated by the ZnO/ZCIS heterojunction was fully maintained.

**Figure 7:**
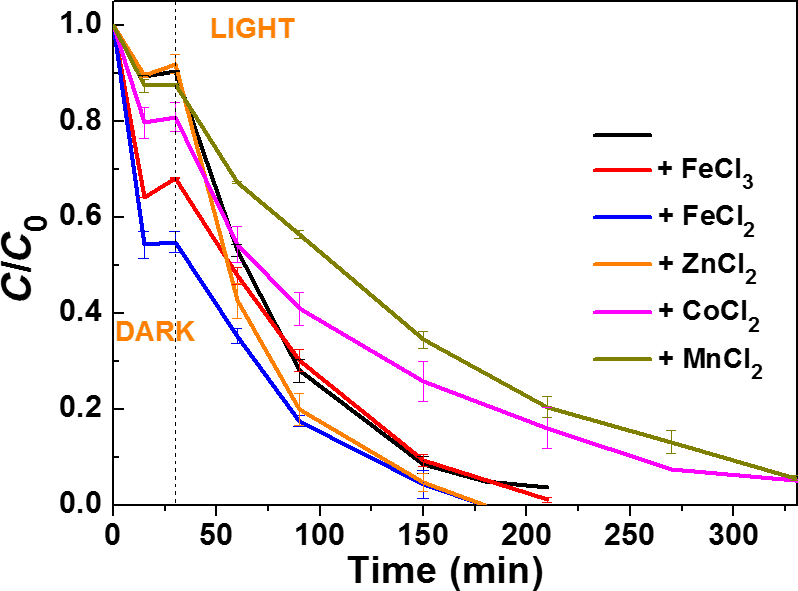
Influence of some transition metal chlorides used at 100 µM concentration on the photocatalytic efficiency of the ZnO/ZCIS catalyst (the black curve corresponds to the reference photocatalytic test).

The effect of transition metal salts (Fe^3+^, Fe^2+^, Zn^2+^, Co^2+^ and Mn^2+^) used at a 100 µM concentration and in their chloride forms was also studied at pH 7.0 (Figure 7). Co^2+^, Fe^3+^ and especially Fe^2+^ markedly affect the dark adsorption of Orange II while Mn^2+^ and Zn^2+^ had almost no influence. We suppose that Co^2+^, Fe^3+^ and Fe^2+^ strongly adsorb at the surface of the ZnO/ZCIS catalyst and thus enhance the adsorption of the Orange II anionic dye via a bridging effect. Only Co^2+^ and Mn^2+^ significantly reduce the photocatalytic rate (*k* = 0.008 min^−1^ both in the presence of Mn^2+^ and Co^2+^). With all other transition metal chlorides, *k* values were higher than that determined for the degradation of Orange II in the absence of salts (*k* = 0.022, 0.021 and 0.024 min^−1^ for reactions conducted in the presence of FeCl_3_, FeCl_2_ and ZnCl_2_, respectively). Noteworthy is also that metal cations in their lowest oxidation state (like Fe^2+^) do not trap holes (Fe^2+^ + h^+^ → Fe^3+^) and that metal cations in their highest oxidation state do not scavenge electrons (Fe^3+^ + e^−^ → Fe^2+^) – contrary to results observed with many TiO_2_-based photocatalysts [49]. We suppose that this result originates from the heterojunction built between the ZnO and ZCIS materials, which improves charge transfer processes between the two materials (for example, fast transfer of electrons from ZCIS QDs to ZnO followed by the reaction of these electrons with O_2_ to generate superoxide anions).

#### Recycling and stability

**Figure 8:**
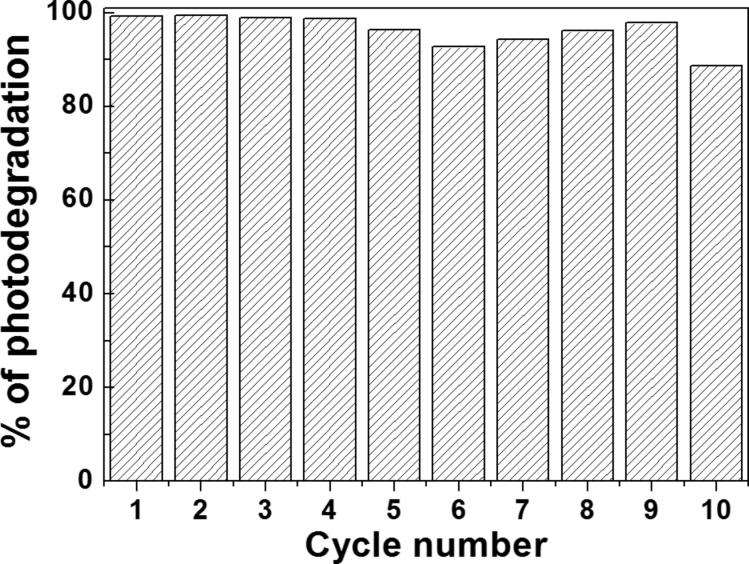
Recyclability of the ZnO/ZCIS photocatalyst.

The recycling of the ZnO/ZCIS photocatalyst was evaluated. For this purpose, once the photodegradation of Orange II was complete, the photocatalyst was recovered by centrifugation and reused without any treatment in the next cycle of photocatalysis. As shown in Figure 8, after ten cycles of Orange II photodegradation, the ZnO/ZCIS catalyst only exhibits a weak decrease in activity (89% of photodegradation after the 10th cycle) thus indicating that it is not photo-corroded during the repeated catalytic experiments and that byproducts do not block the active sites at its surface. SEM and XRD were further used to examine the stability of the photocatalyst (Supporting Information File 1, Figure S9). No obvious changes in crystallinity and morphology were detected, indicating its good stability.

#### Mechanism

**Figure 9:**
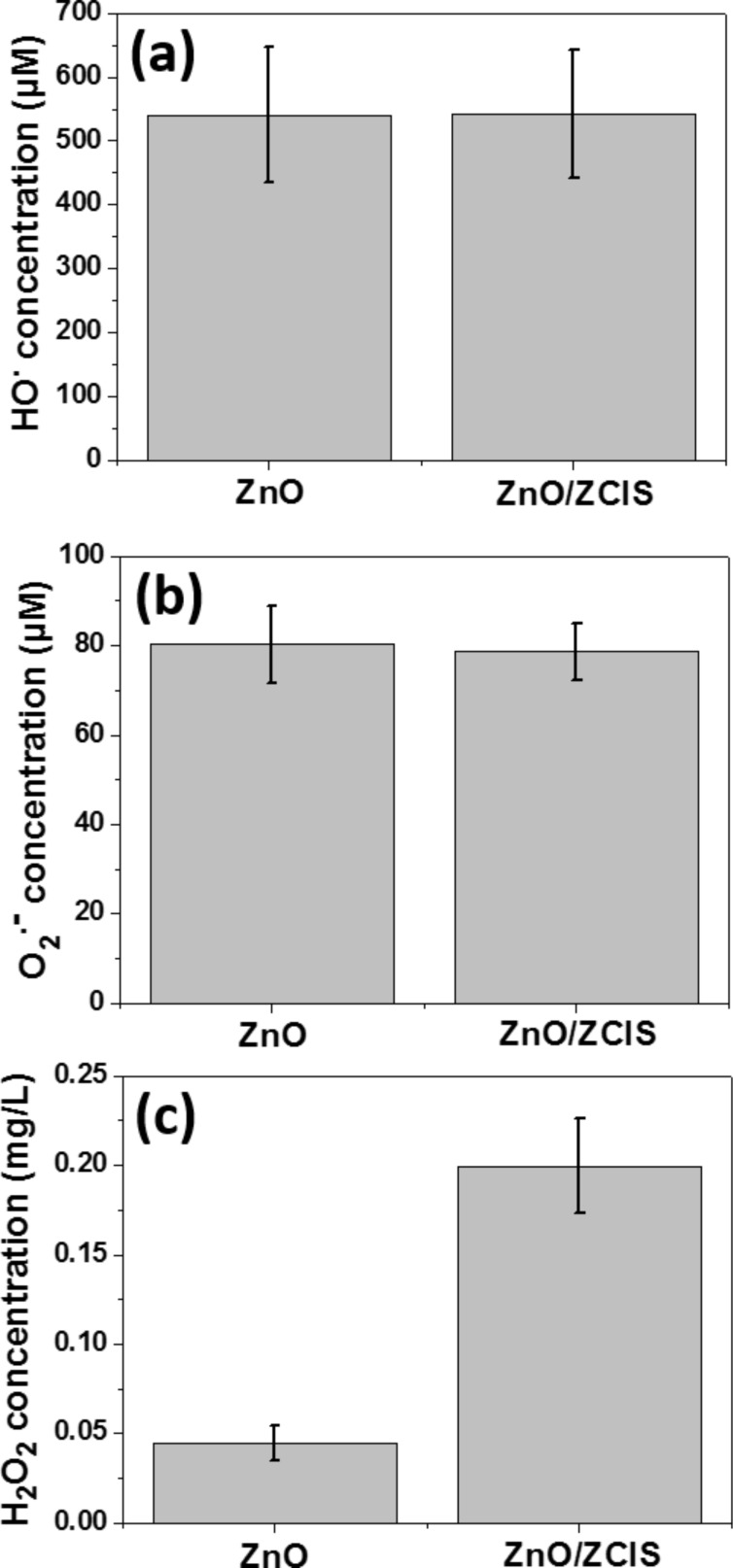
Concentration of (a) hydroxyl and (b) superoxide radicals and (c) hydrogen peroxide produced by ZnO and ZnO/ZCIS particles under irradiation using a Hg–Xe lamp. Concentrations of hydroxyl and superoxide radicals and hydrogen peroxide were determined after 30 min, 1 h and 45 min irradiation, respectively.

The photocatalytic ROS production by the ZnO and ZnO/ZCIS materials was evaluated using selective spectroscopic probes. In a first set of experiments, disodium terephthalate (DST), nitroblue tetrazolium (NBT) and crystal violet (CV)/horse radish peroxidase (HRP) were used to detect and quantify the production of ^•^OH and O_2_^•−^ radicals and H_2_O_2_, respectively [42]. In control experiments, no ROS production was detected when ZnO and ZnO/ZCIS particles were mixed with these probes under dark conditions for 1 h. Under Hg–Xe lamp irradiation, all probes responded positively, indicating that ZnO and ZnO/ZCIS particles are able to produce ^•^OH, O_2_^•−^ radicals and H_2_O_2_. After 1 h of irradiation, the ZnO and ZnO/ZCIS materials were found to produce similar amounts of ^•^OH and O_2_^•−^ radicals ([^•^OH] = 550 µM and [O_2_^•−^] = 80 µM) (Figure 9a,b), indicating that these species are not responsible for the enhanced photocatalytic activity of the ZnO/ZCIS composite compared to ZnO. A markedly increased production of H_2_O_2_ by ca. 4-fold is observed for the ZnO/ZCIS composite (Figure 9c). After 45 min of irradiation, the ZnO and ZnO/ZCIS photocatalysts generated ≈0.05 and 0.20 mg/L H_2_O_2_, respectively. This result also shows that H_2_O_2_ accumulated during photocatalytic reactions conducted with the ZnO/ZCIS particles and is not decomposed into ^•^OH radicals under simulated solar light irradiation.

**Figure 10:**
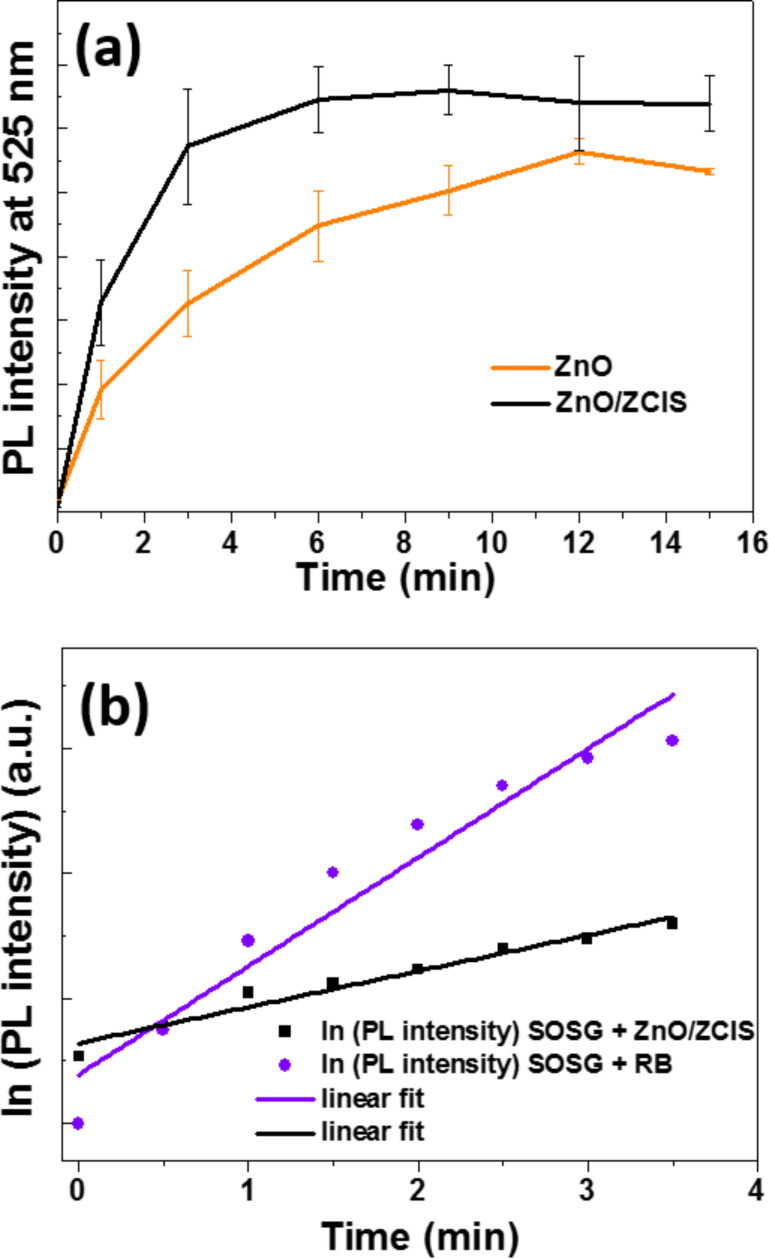
(a) Time evolution of SOSG−endoperoxide (SOSG-EP) photoluminescence (PL) intensity upon irradiation with a Hg–Xe lamp (intensity = 50 mW/cm^2^) of the ZnO/ZCIS composite and of ZnO. (b) Linear fit of the plots of ln(PL intensity) of SOSG-EP.

Singlet molecular oxygen ^1^O_2_ generation has scarcely been reported for photocatalysts and may originate from the oxidation of O_2_^•−^ into ^1^O_2_ and triplet oxygen ^3^O_2_ or from the energy transfer of the photo-excited catalyst to ^3^O_2_ [50–53]. ^1^O_2_ has been demonstrated to play a key role in some TiO_2_-catalyzed photodegradation schemes [54–55]. We evaluated the production of ^1^O_2_ by ZnO and ZnO/ZCIS materials using the specific SOSG fluorescent probe [42,56–57]. The fluorescence of SOSG−endoperoxide (SOSG-EP) formed through the addition of ^1^O_2_ on the anthracene unit of SOSG was monitored at ≈525 nm (Figure 10a). An increased production of ^1^O_2_ for the ZnO/ZCIS catalyst compared to ZnO was observed, especially during the first ten minutes of irradiation. The kinetic of SOSG-EP formation was used for the determination of the ^1^O_2_ quantum yield (Ф^1^O_2_) of ZnO/ZCIS particles. Rose Bengal (RB) was used as a reference photosensitizer (Ф^1^O_2_ (RB) = 75% in water) [58]. The plots ln(PL_525_) of SOSG-EP vs time for RB and for the ZnO/ZCIS composite show a good linear fit during the first 3.5 min of irradiation (Figure 10b). The Ф^1^O_2_ of the ZnO/ZCIS catalyst was estimated using the equation: Ф^1^O_2_ (ZnO/ZCIS) = Ф^1^O_2_ (RB) × (*k*_ZnO/ZCIS_/*k*_RB_), where *k*_RB_ and *k*_ZnO/ZCIS_ were determined from the slopes of the PL increase of SOSG-EP over time, expressed as d(ln(PL_0_/PL_t_))/d(*t*), of RB and ZnO/ZCIS particles, respectively, and was found to be 24.8%.

**Figure 11:**
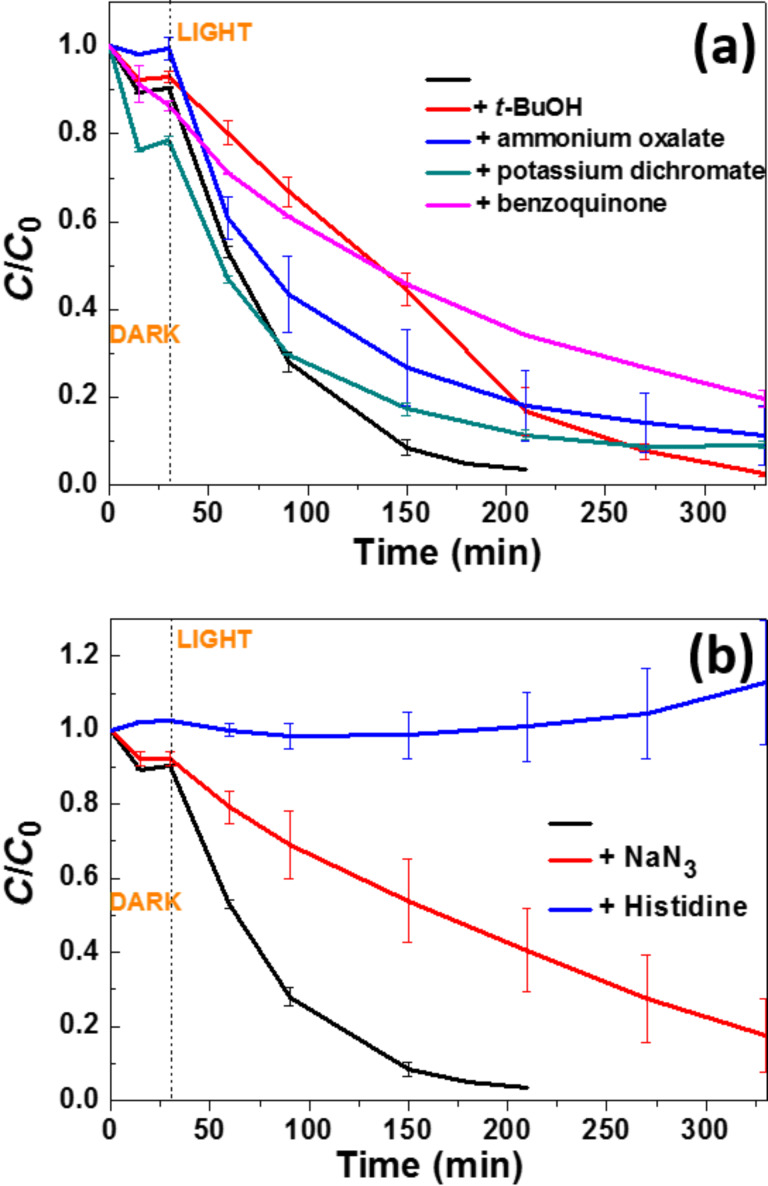
(a) Influence of ^•^OH, O_2_^•−^, electron and hole scavengers and (b) influence of ^1^O_2_ scavengers on the photocatalytic activity of the ZnO/ZCIS composite. The black line corresponds to the experiment conducted without a scavenger.

To confirm the key role of some of these ROS in the photodegradation mechanism mediated by the ZnO/ZCIS catalyst, we first investigated the effect of dissolved O_2_ by purging the Orange II solution with N_2_. In the N_2_-saturated solution, the photodegradation rate was decreased compared to air-equilibrated solutions, which shows that O_2_ is significant for the Orange II oxidation mediated by the ZnO/ZCIS photocatalyst (Supporting Information File 1, Figure S10). We also conducted photocatalytic experiments in the presence of *t*-BuOH, ammonium oxalate, potassium dichromate and benzoquinone used as ^•^OH, hole, electron, and O_2_^•−^ scavengers, respectively. As can be seen from Figure 11a, the addition of *t-*BuOH (10 mL/L) and benzoquinone (10 mg/L) had the more deleterious effects on the photodegradation rate compared to the experiment conducted without scavenger, thus indicating that ^•^OH and O_2_^•−^ play an important role in the oxidation process of the dye. The inhibiting effects of ammonium oxalate (1 g/L) and potassium dichromate (100 mg/L) were less pronounced, suggesting that the direct oxidation of Orange II by the holes only played a minor role in the degradation mechanism and that photo-generated electrons were efficiently captured by dissolved O_2_ molecules. The key role played by ^1^O_2_ in the photodegradation was demonstrated by adding in the reaction medium NaN_3_ (1 g/L), a physical quencher of ^1^O_2_ [59], or histidine (1 g/L), both able to react with ^1^O_2_ at high rates (Figure 11b) [60–61]. The results obtained show that the degradation rate was markedly reduced when photocatalytic experiments were conducted in the presence of NaN_3_ and completely inhibited when using histidine.

**Figure 12:**
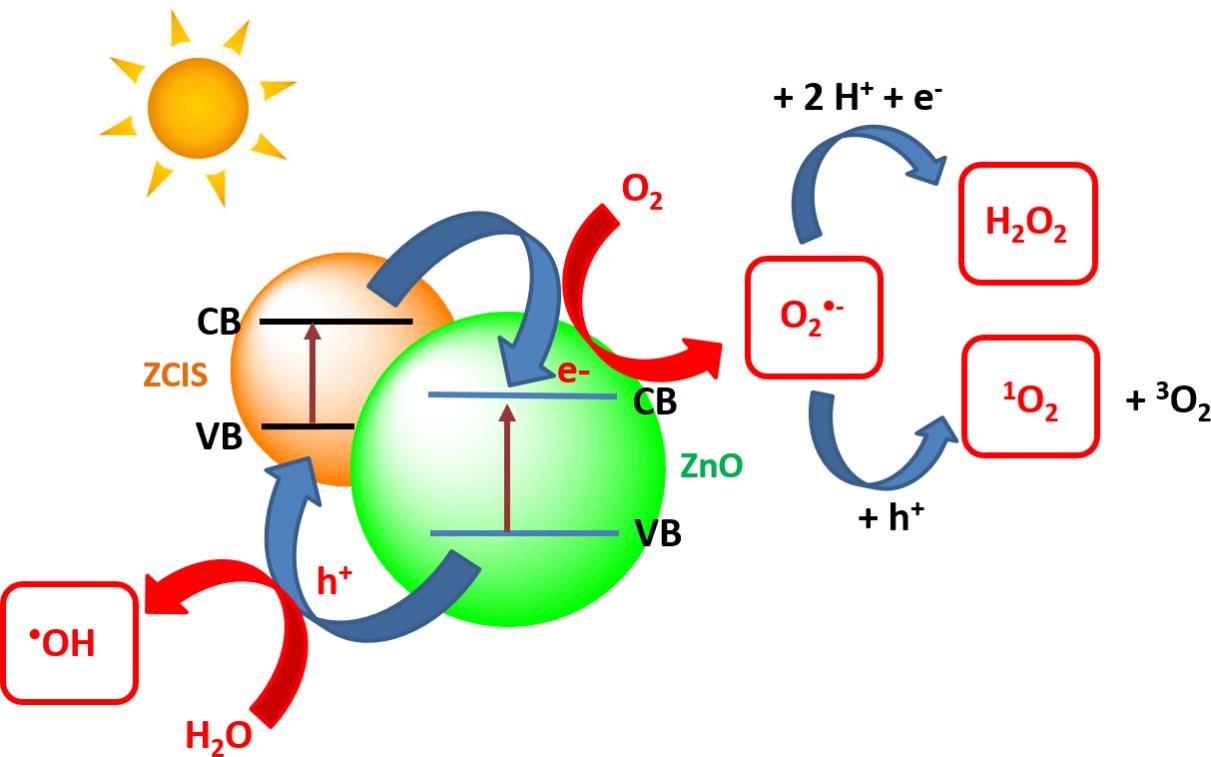
Schematic illustration of the charge transfer process and of the ROS production in the ZnO/ZCIS photocatalyst.

These experiments confirm that the ZnO/ZCIS composite has an enhanced ability to produce H_2_O_2_ and ^1^O_2_ compared to ZnO and that ^1^O_2_ plays a key role in the photodegradation mediated by the ZnO/ZCIS composite. Upon simulated solar light irradiation of the ZnO/ZCIS catalyst, electrons are promoted from the VB to the CB of both ZCIS and ZnO materials (although ZCIS QDs are more easily excited by the light source used in this study). Because the CB of ZnO is more positive than that of the ZCIS QDs (−0.335 eV and −0.59 eV vs NHE for ZnO and ZCIS, respectively, see Figure 4), the excited electrons in ZCIS QDs are probably quickly transferred to the CB of ZnO due to the electrostatic field at the junction. Meanwhile, the holes in the VB of ZnO are transferred to the VB of ZCIS QDs (Figure 12). These transfers result in an efficient electron/hole separation and thus in a prolonged lifetime of the charge carriers due to the extended path. The separated electrons and holes can then initiate reduction and oxidation reactions with oxygen and water adsorbed on the catalyst surface leading to an enhanced photocatalytic activity. The positions of the VB and CB of ZnO and ZCIS semiconductors determine the type of ROS produced during the photocatalysis. The redox potentials of O_2_^•−^/O_2_ and H_2_O/^•^OH couples are −0.16 eV and 2.32 eV vs NHE at pH 7, respectively. To generate O_2_^•−^, photogenerated electrons must have a potential less than −0.16 eV while photogenerated holes must have a potential greater than 2.32 eV to produce ^•^OH (Figure 4). These conditions are fulfilled in the ZnO/ZCIS photocatalyst. ZnO and ZCIS materials can both produce O_2_^•−^ radicals but only ZnO is able to generate ^•^OH. Because the free radical half-time of O_2_^•−^ is much higher (up to one microsecond) than that of ^•^OH radicals (one nanosecond) [62–63] and that the ZnO/ZCIS catalyst has a higher ability to generate O_2_^•−^ by reduction of O_2_ adsorbed at his surface than ^•^OH radicals, we privilege the mechanism depicted in Figure 12 to explain the enhanced photocatalytic activity of ZnO/ZCIS compared to ZnO. Processes like disproportionation of O_2_^•−^ (2O_2_^•−^ + 2H^+^ → O_2_ + H_2_O_2_) and protonation of O_2_^•−^ into hydroperoxyl HO_2_^•^ followed by reduction (O_2_^•−^ + 2 H^+^ + e^−^ → H_2_O_2_) may account for the high production of H_2_O_2_. Because high amounts of O_2_^•-^ radicals are formed by the ZnO/ZCIS catalyst, we suppose that ^1^O_2_ is preferentially generated by the oxidation of O_2_^•−^ by holes rather than by an energy transfer of the photo-excited catalyst to ^3^O_2_.

## Conclusion

In summary, we have successfully prepared ZnO/ZCIS composites via a facile thermal treatment at 400 °C for 15 min. Compared to ZnO nanoparticles, the ZnO/ZCIS composite prepared with a 40:1 ZnO/ZCIS weight ratio displays enhanced photocatalytic capability for the degradation of Orange II dye under simulated solar light irradiation. The photocatalytic mechanism investigations demonstrate that singlet oxygen and hydrogen peroxide play a key role in the degradation of the dye. The improved solar light photocatalytic activity of the ZnO/ZCIS composite is achieved by the increased lifetime of charge carrier transfer and by the increased light absorption in the visible region due to the heterojunction created between the ZCIS QDs and ZnO nanoparticle. Finally, due to its ease of preparation, low cost, high stability and weak sensitivity to interfering species such as salts, the ZnO/ZCIS photocatalyst exhibits a high potential for practical applications beyond the degradation of organic pollutants, for example, for the production of new energy sources.

## Supporting Information

The Supporting Information contains UV–vis absorption, photoluminescence emission and diffuse reflectance spectra, Tauc plots, EDS analysis, XPS spectra, plots of ln(*C*_0_/*C*), an XRD pattern and an SEM image of the reused catalyst and the photocatalytic experiments in air-equilibrated and N_2_-purged aqueous solutions.

File 1 Additional figures.
