# Supplementary material for: ZnO nanoparticles sensitized by CuInZnxS2+x quantum dots as highly efficient solar light driven photocatalysts
Source: Beilstein J Nanotechnol. 2017 May 17;8:1080–93. doi: 10.3762/bjnano.8.110 (PMC5480363; doi:10.3762/bjnano.8.110)
Supplement: File 1 — Additional figures. [file Beilstein_J_Nanotechnol-08-1080-s001.pdf]

Supporting Information
for

ZnO nanoparticles sensitized by $CuInZn_xS_{2+x}$ quantum dots as highly efficient solar light driven photocatalysts

Florian Donat[1], Serge Corbel[1], Halima Alem[2], Steve Pontvianne[1], Lavinia Balan[3], Ghouti Medjahdi[2] and Raphaël Schneider*[1]

Address: [1]CNRS and Université de Lorraine, Laboratoire Réactions et Génie des Procédés (LRGP), CNRS UMR 7274, 1 rue Grandville 54001 Nancy, France, [2]CNRS and Université de Lorraine, Institut Jean Lamour (IJL), UMR CNRS 7198, BP 70239, 54506 Vandoeuvre-lès-Nancy Cedex, France and [3]Institut de Science des Matériaux de Mulhouse (IS2M), CNRS UMR 7361, 15 rue Jean Starcky, 68093 Mulhouse, France

Email: Raphaël Schneider - raphael.schneider@univ-lorraine.fr

* Corresponding author

Additional figures

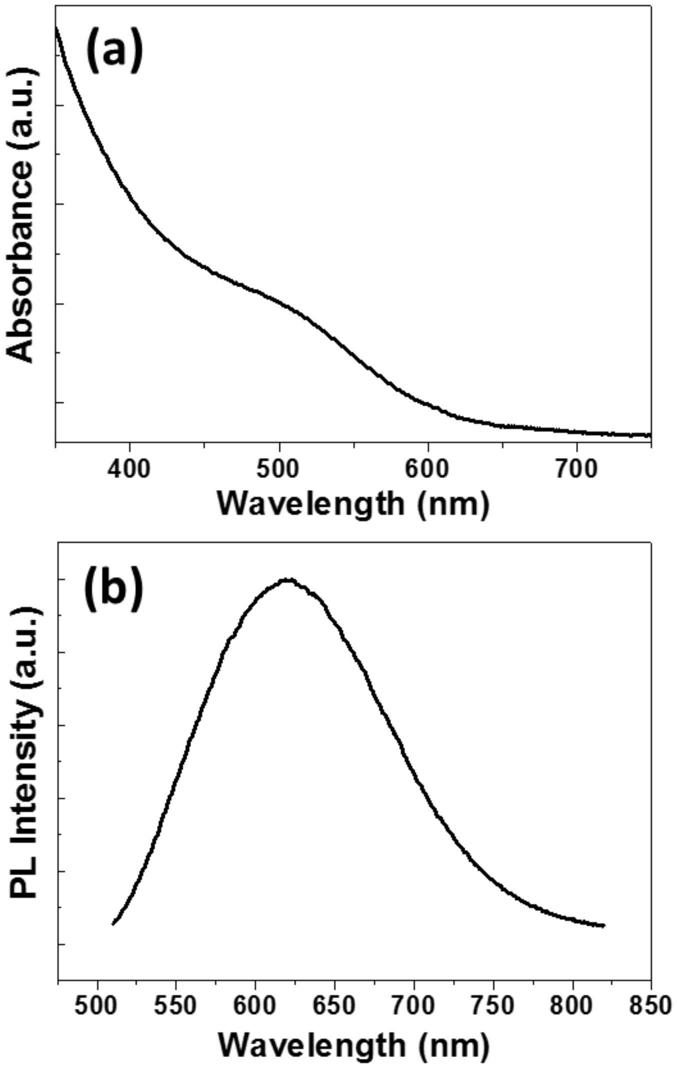

Figure S1: (a) UV–visible absorption and (b) PL emission spectra of ZCIS QDs (λ_{ex} = 500 nm).

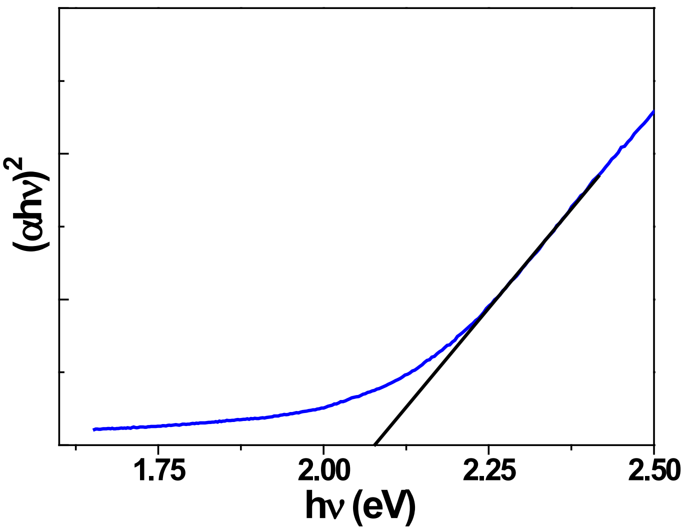

Figure S2: Tauc plot of ZCIS QDs.

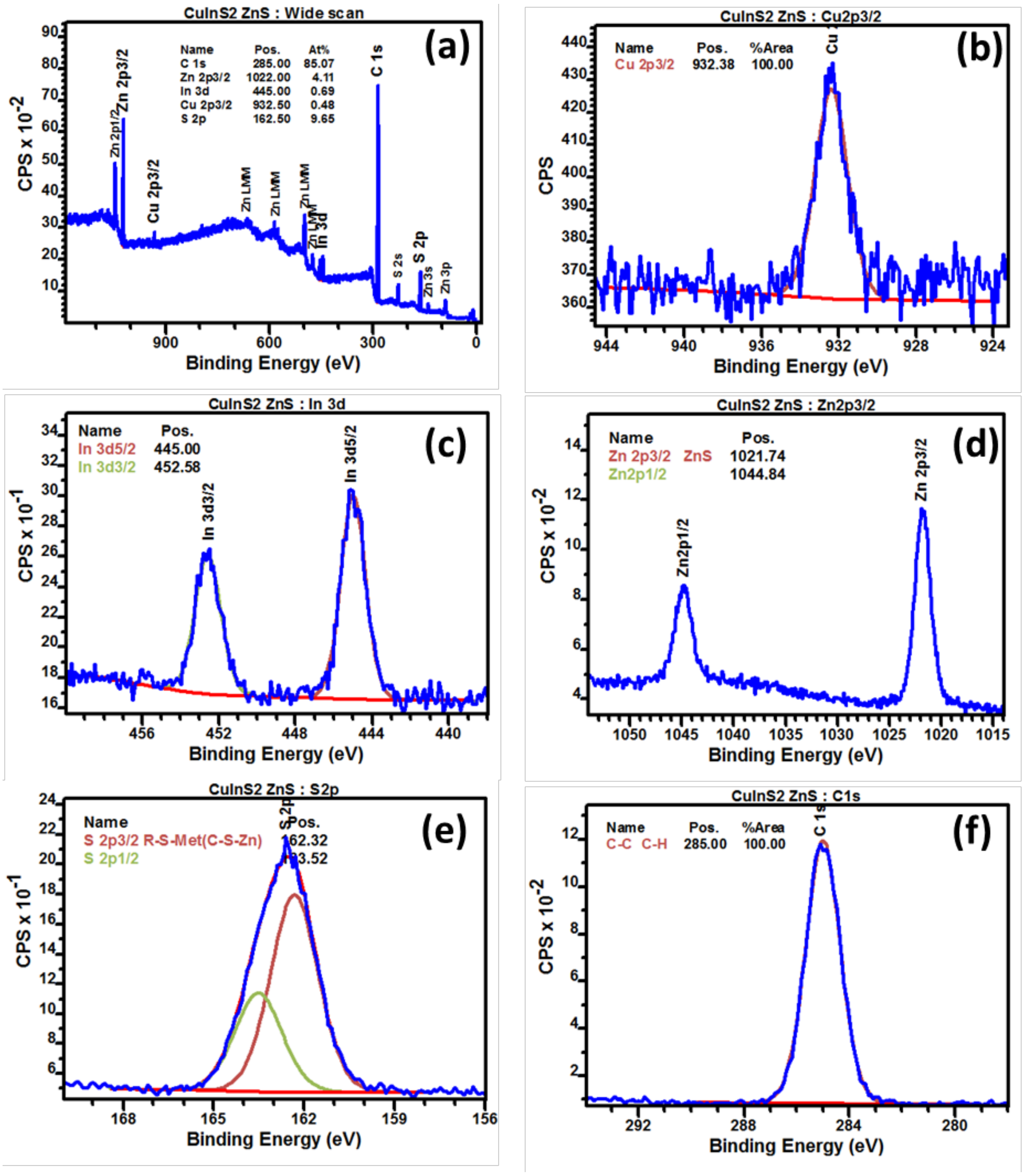

Figure S3: Survey (a), Cu 3d (b), In 3d (c), Zn 2p (d), S 2p (e) and C 1s (f) XPS spectra of DDT-capped ZCIS QDs.

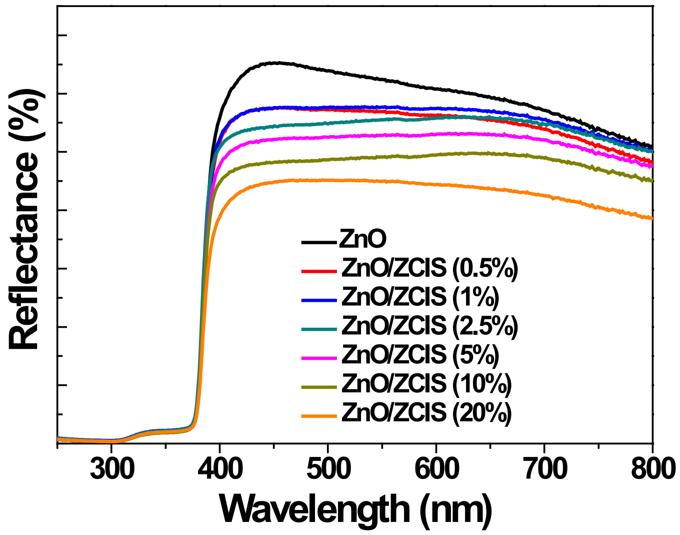

Figure S4: UV–visible diffuse reflectance spectra of ZnO and ZnO/ZCIS composites when varying the ZCIS loading from 0 to 20% (ZnO/ZCIS composites were prepared by heating ZnO particles and ZCIS QDs at 400 °C for 15 min).

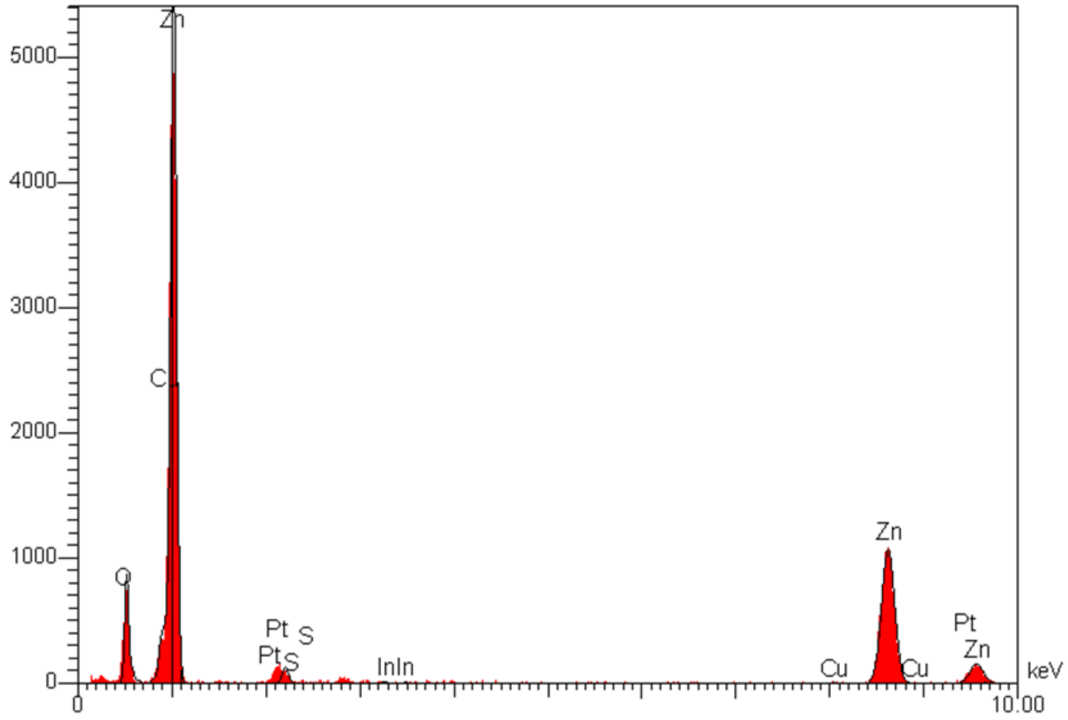

Figure S5: Typical EDS analysis of the ZnO/ZCIS photocatalyst.

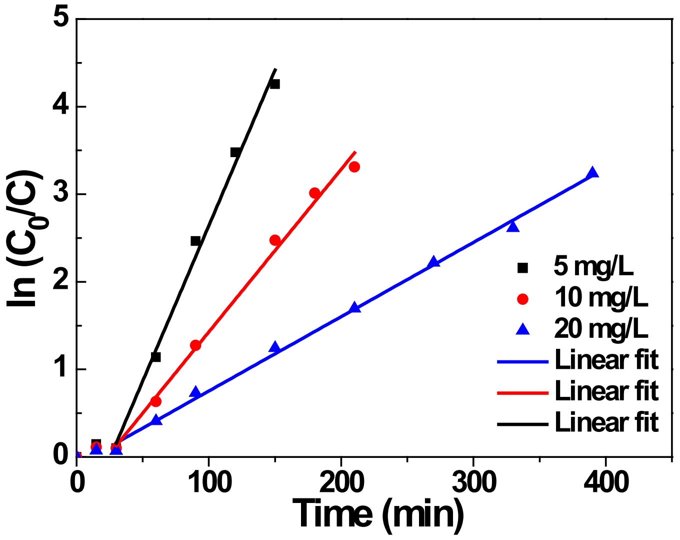

Figure S6: Plots of ln(C_0/C) of Orange II versus reaction time when varying the dye concentration (5, 10 or 20 mg/L).

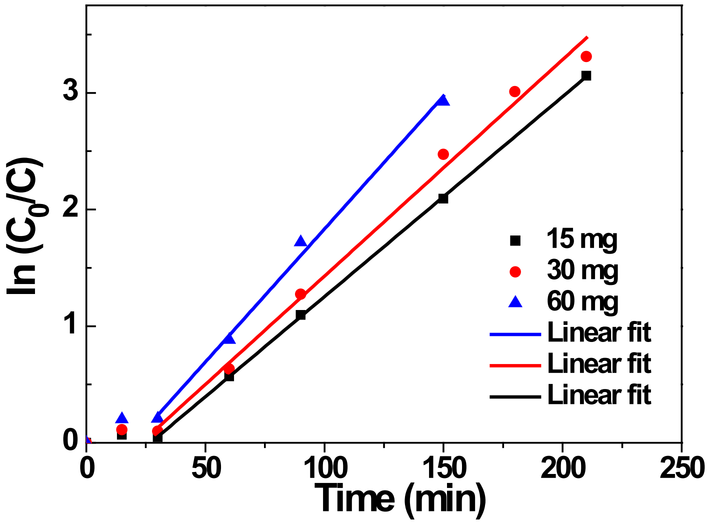

Figure S7: Plots of ln(C_0/C) of Orange II versus reaction time when varying the amount of catalyst (15, 30 or 60 mg of ZnO/ZCIS and concentration of Orange II = 10 mg/L).

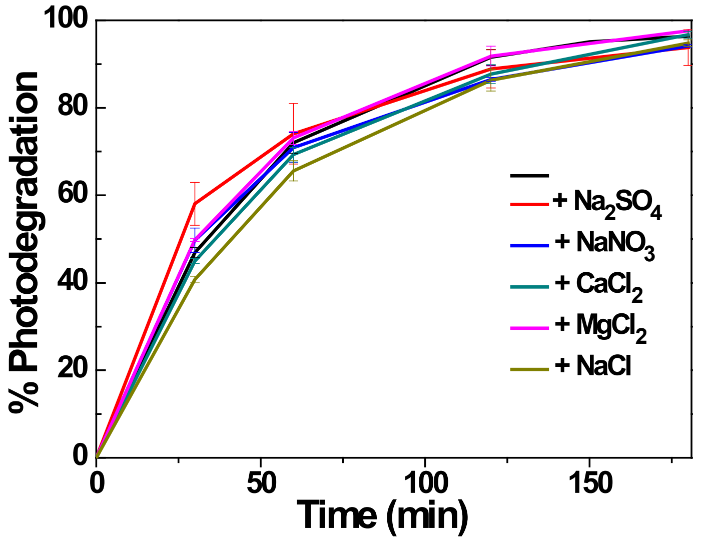

Figure S8: Influence of Na_2SO_4, $NaNO_3$, $CaCl_2$, $MgCl_2$ and NaCl (used at a 10 mM concentration) on the photocatalytic activity of the ZnO/ZCIS composite.

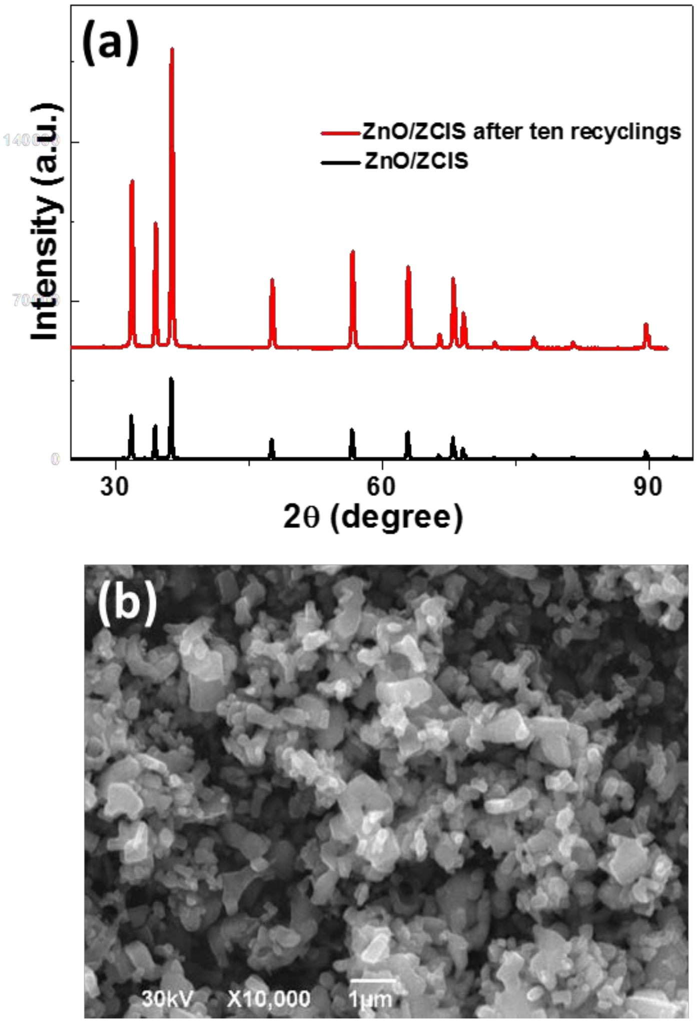

Figure S9: (a) XRD patterns of the ZnO/ZCIS photocatalyst after synthesis (black line) and after ten reuses (red line) and (b) SEM image of the photocatalyst after ten reuses.

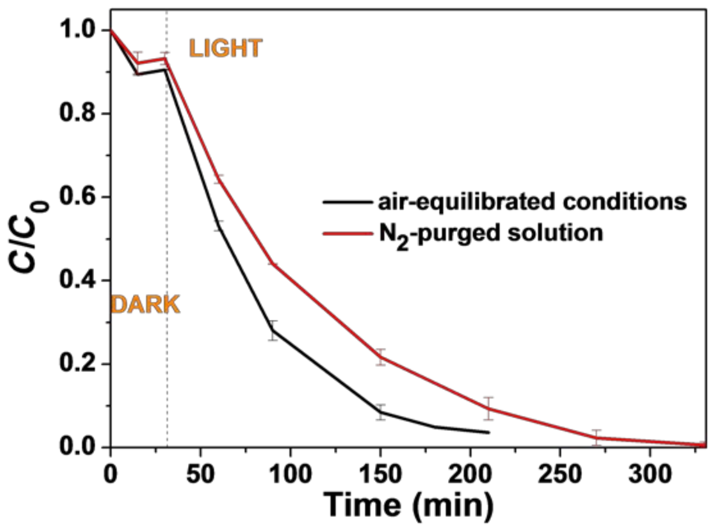

Figure S10: Photocatalytic degradation of Orange II in air-equilibrated and N_2-purged aqueous solutions.
